# Warmer temperatures reduce the vectorial capacity of malaria mosquitoes

**DOI:** 10.1098/rsbl.2011.1075

**Published:** 2011-12-21

**Authors:** Krijn P. Paaijmans, Simon Blanford, Brian H. K. Chan, Matthew B. Thomas

**Affiliations:** 1 Center for Infectious Disease Dynamics, Pennsylvania State University, University Park, PA 16802, USA; 2 Department of Entomology, Pennsylvania State University, University Park, PA 16802, USA; 3 Department of Biology, Pennsylvania State University, University Park, PA 16802, USA

**Keywords:** parasite infection, pathogen transmission, malaria risk, climate change, extrinsic incubation period, mortality

## Abstract

The development rate of parasites and pathogens within vectors typically increases with temperature. Accordingly, transmission intensity is generally assumed to be higher under warmer conditions. However, development is only one component of parasite/pathogen life history and there has been little research exploring the temperature sensitivity of other traits that contribute to transmission intensity. Here, using a rodent malaria, we show that vector competence (the maximum proportion of infectious mosquitoes, which implicitly includes parasite survival across the incubation period) tails off at higher temperatures, even though parasite development rate increases. We also show that the standard measure of the parasite incubation period (i.e. time until the first mosquitoes within a cohort become infectious following an infected blood-meal) is incomplete because parasite development follows a cumulative distribution, which itself varies with temperature. Including these effects in a simple model dramatically alters estimates of transmission intensity and reduces the optimum temperature for transmission. These results highlight the need to understand the interactive effects of environmental temperature on multiple host-disease life-history traits and challenge the assumptions of many current disease models that ignore this complexity.

## 1. Introduction

The ecology of many vector–parasite/pathogen interactions is strongly influenced by environmental temperature [1]. Accordingly, it has been suggested that the dynamics and distribution of a range of vector-borne diseases, including malaria, dengue, viral encephalitis, schistosomiasis, Lyme disease and West Nile virus, could be impacted by climate change [1,2].

Predicting the extent of possible changes in disease patterns requires detailed understanding of how a suite of vector and parasite traits respond to temperature. However, in many cases the nature of the temperature-dependent relationships remains poorly defined. For example, vector competence, which describes the ability of a vector to acquire, maintain and transmit a parasite/pathogen, is widely assumed to be temperature-insensitive. Yet, evidence from a limited number of studies indicates that vector competence can change with temperature [3–6].

Similarly, the standard degree-day models used to characterize the development of parasites/pathogens within the vector (defined as the extrinsic incubation period, or EIP) typically give a single value per temperature. During the EIP, pathogens go through very many replication cycles before migrating to the salivary glands where they can be transmitted to humans. The number of infectious mosquitoes (i.e. with pathogens in the salivary glands) in a mosquito cohort is expected to increase, from zero to the maximum number observed, over several days [7,8]. It is unclear how this distribution is affected by temperature, or how selecting the starting point, the median or endpoint of the distribution alters estimates of transmission intensity.

Here, using the rodent malaria *Plasmodium yoelii* and the Asian malaria vector *Anopheles stephensi*, we examine these standard assumptions and explore the implications for our understanding of the effects of temperature on disease transmission.

## 2. Material and methods

Twenty-five mice (female C57Bl/6 laboratory mice, Charles River Laboratories) were inoculated with 10^5^ *P. yoelii* parasites (clone 17XNL, from the WHO Registry of Standard Malaria Parasites, University of Edinburgh, UK). Four days after inoculation, approximately 2000 female *A. stephensi* mosquitoes (2–4 days old) were pooled in a single large cage and allowed to feed for 30 min on the anaesthetized mice. Post blood-feed, the females that took a full blood-meal were randomly distributed among four incubators (two cages per incubator) maintained at 20°C, 22°C, 24°C (the standard temperature for *P. yoelii* transmission) and 26°C ± 1°C, with 90 ± 5% relative humidity and 12 L : 12 D cycle photoperiod. Mosquitoes were fed *ad libitum* on 10 per cent glucose solution supplemented with 0.05 per cent paraaminobenzoic acid. Two and three days post blood-feed, mosquitoes were provided with egg laying bowls.

Mosquito salivary glands were dissected under a standard dissecting microscope, with 25 mosquitoes (randomly selected from the two cages) per temperature treatment per time-interval. We recorded if a mosquito harboured sporozoites in the salivary glands (hereafter referred to as ‘infectious mosquito’). During the first dissection time-point, midguts were also dissected to establish baseline malaria infection prevalence, i.e. the proportion of females with oocysts on their midgut. The number of oocysts per midgut was also recorded for each temperature treatment (square root transformed to meet normality assumptions). Daily inspection of the oocyst development for a small number of mosquitoes gave an indication of when oocysts were about to complete their development. Salivary glands were dissected on that day, and on at least 5 subsequent days to capture the cumulative sporozoite release. Additional mosquitoes were dissected over the subsequent one to two weeks to verify that maximum prevalence of infectious mosquitoes had been reached (hereafter, referred to as ‘maximum transmission prevalence’).

The cumulative change in proportion of infectious mosquitoes (*b*) over time was described by a logistic model *b* = *b*_max_/(1 + e^−*k*(*t*–*t*_m_^^)^), where *b*_max_ is the upper asymptote (or the maximum transmission prevalence), *t*_*m*_ a constant and *k* the time at which the absolute increase in *b* is maximal. Best-fit logistic functions were used to estimate the maximum transmission prevalence, together with the EIP_10_, EIP_50_ and EIP_90_ (i.e. the time to 10, 50 and 90% of the maximum prevalence) at a given temperature. To test if maximum infection prevalence varied significantly across temperature, we considered data points greater than EIP_90_.

Transmission intensity was characterized using the standard formulation for vectorial capacity (*C*) for a single host–pathogen–vector system [9]:
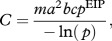
where *m* is the vector : human ratio; *a* vector biting frequency; *p* daily vector survival rate; *bc*, vector competence (a combination of *b*, the probability of a person becoming infected via a bite from an infectious vector, and *c*, the probability of a vector becoming infected by feeding on an infectious person); and EIP. Effects of temperature were explored using the empirical measures of *b* (maximum transmission prevalence), *c* (baseline infection prevalence) and EIP. There is very little information on the effects of temperature on the number of mosquito vectors per human so we set *m* arbitrarily to 1. Temperature has minimal effect on adult mosquito survival across the range of experimental temperatures explored [10]. However, since survival can vary substantially between malaria vector species [11] we chose two representative daily survival probabilities (0.8 and 0.9). Biting frequency, *a*, was described using the temperature-dependent function of Lardeux *et al*. [12].

## 3. Results

**Figure 1.**
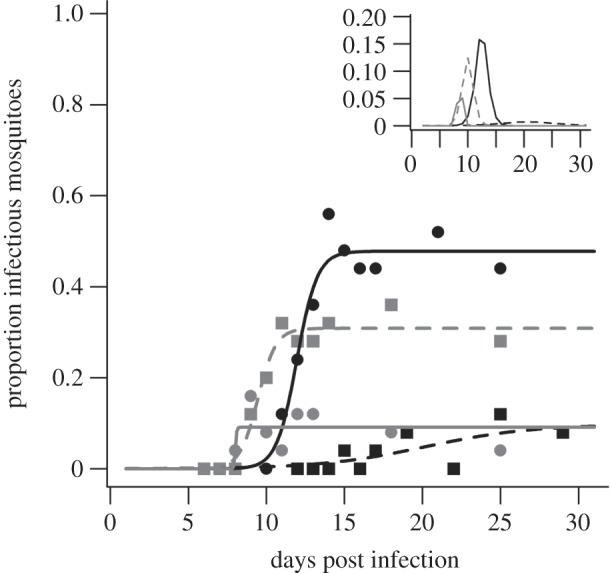
Parasite development rates and vector competence over time at four temperatures (black squares, black dashed line, 20°C; black circles, black solid line, 22°C; grey squares, grey dashed line, 24°C; grey circles, grey solid line, 26°C). The data points indicate salivary infection prevalence at particular dissection times for the different temperature treatments. The lines represent best-fit logistic growth curves for each temperature as described in table 1. Inset shows the daily proportion of new infectious individuals in a mosquito cohort at the four different temperatures.

Temperature did not affect baseline infection prevalence (mean of 91%; *χ*^2^ = 3.57, d.f. = 3, *p* = 0.312, hence *c* equals 0.91), nor the mean number of oocysts per midgut (221 ± 16; *F*_3,90_ = 1.96, *p* = 0.125). Increasing temperature did, however, increase parasite development rate (1/EIP), with the first infectious mosquitoes observed at days 15, 11, 9 and 8 at 20°C, 22°C, 24°C and 26°C, respectively (figure 1). Warmer temperatures also reduced the standard deviation in EIP among mosquitoes (inset figure 1 and table 1).

**Table 1.** Parameters of the logistic growth model (and 95% CI) used to characterize the cumulative distribution of the number of mosquitoes that become infectious (*b*) over time at different temperatures. Parasite development times (EIP, in days) and proportion of infectious mosquitoes across temperature, when 10, 50 and 90% of the final infectious population is infectious are also shown.

	20°C	22°C	24°C	26°C
*b*_max_	9.7 (−6.5–26.0)	47.8 (46.7–49.0)	30.9 (29.6–32.1)	9.1 (5.0–13.3)
*t*_*m*_	20.2 (19.4–21.0)	11.9 (6.5–17.4)	9.4 (5.9–12.9)	8.0 (−1.7E7–1.7E7)
*k*	0.3 (−14.7–15.3)	1.5 (1.0–2.1)	1.7 (1.2–2.2)	24.1 (−3.8E10–3.8E10)
*R*^2^	0.501	0.954	0.962	0.539
EIP_10_	12.8	10.5	8.2	7.9
EIP_50_	20.2	11.9	9.4	8.0
EIP_90_	27.6	13.4	10.7	8.1
*b*_10_	0.010	0.048	0.031	0.009
*b*_50_	0.049	0.239	0.154	0.046
*b*_90_	0.088	0.430	0.278	0.082

In contrast to development rates, increasing temperature did not simply increase vector competence. Maximum transmission prevalence was observed at 22°C (48%; figure 1 and table 1), and this prevalence was significantly higher than that at 24°C (*χ*^2^ = 9.44, d.f. = 1, *p* = 0.002). Prevalence at 24°C, in turn, was higher than that at 26°C (*χ*^2^ = 24.24, d.f. = 1, *p* < 0.001). The proportion of infectious mosquitoes was clearly reduced at 20°C but did not plateau by day 30 so was not analysed further.

**Figure 2.**
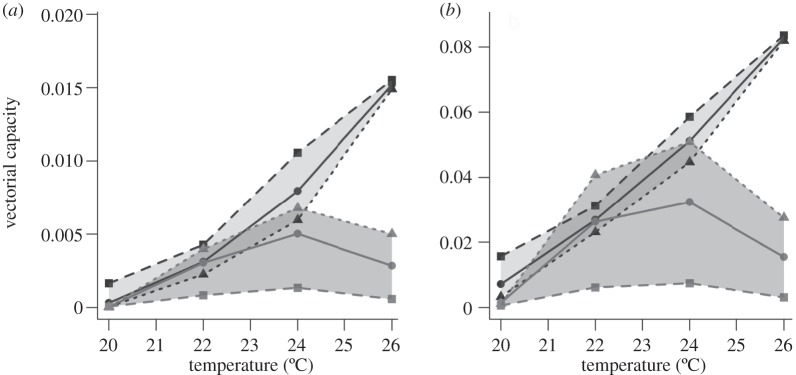
Mosquito vectorial capacity across temperature. Vectorial capacity estimated using either temperature-independent (black data points) or temperature-dependent (grey data points) measures of vector competence, and the EIP_10_ (squares), EIP_50_ (circles) or EIP_90_ (triangles; i.e. the time to 10, 50 and 90% of the maximum prevalence at a given temperature) for a daily mosquito survival probability of (*a*) 0.8 and (*b*) 0.9.

The conventional approach of assuming temperature-independent vector competence (whereby *b* = 0.24, the mean empirical value across temperature, and *c* = 0.91) predicted vectorial capacity to be highest at 26°C (figure 2). At this temperature, whether EIP was defined at the 10, 50 or 90th percentile of the distribution had little effect. At cooler temperatures, however, selecting the EIP_50_ or EIP_90_ reduced estimates of *C* relative to the EIP_10_; the time taken to reach the much longer EIP_50_ or EIP_90_ at cooler temperatures means there are fewer mosquitoes alive to transmit.

Allowing temperature-dependent variation in vector competence changed these patterns considerably (figure 2). Maximum *C* is now predicted at 24°C, regardless of the daily mosquito survival probability, and exhibits a strong decline at lower and higher temperatures (i.e. compared with conventional estimates vectorial capacity is reduced 5.0-, 1-, 1.6- and 5.3-fold, at 20°C, 22°C, 24°C and 26°C, respectively). Moreover, in contrast to the standard predictions, use of EIP_50_ or EIP_90_ increased the estimates of *C* compared with those based on EIP_10_; although it takes longer for 50 or 90 per cent of the infected mosquito population to become infectious, the much higher probability of being infectious at these later time points more than compensates for the increased cumulative daily mortality.

## 4. Discussion

This study demonstrates marked effects of temperature on malaria parasite development rate and vector competence. As expected, warmer temperatures reduce the EIP. In addition, for a given ambient temperature the standard deviation of the EIP decreases as temperature (development rate) increases. We find that whether EIP is defined at an early (EIP_10_) or late (EIP_90_) stage of this distribution can have substantial impact on estimates of vectorial capacity. The observed effects of temperature on vector competence add further complexity. The strongly nonlinear relationship reduces the optimum temperature for transmission (vector competence trades-off against parasite development rate), and also reverses the relative influence of the EIP distribution for estimating vectorial capacity.

Our experiments used a rodent malaria and one species of mosquito and there is clearly a need to extend investigations to human malaria species and to other important vectors [13]. Nonetheless, the *A. stephensi–P. yoelii* system is considered a biologically realistic model [14] and there is no reason to believe that the temperature sensitivity of vector competence and the cumulative distribution in parasite development times are unique to this system.

The mechanisms behind the reduced vector competence at higher temperatures require further investigation but could include direct parasite mortality [15], reduced salivary gland invasion efficiency and sporozoite chemotaxis [16], and/or increased mosquito immune-related responses [17]. In addition, the transmission potential of individual mosquitoes across the EIP range needs to be determined as it is unclear whether mosquitoes at the EIP_10_ and the EIP_90_, for example, are equally infectious to a vertebrate host. Furthermore, our simplifying assumption setting the vector : host ratio (*m*) to 1 overlooks potentially complex effects of temperature on diverse mosquito traits such as immature development and survival, adult survival and fecundity. Adding these effects could further alter vectorial capacity and suggests a need for additional experimentation, ideally under field conditions.

Overall, our results challenge current understanding of the effects of temperature on malaria transmission dynamics. We expect the effects to be robust across human malarias and possibly other vector-borne diseases. If so, the findings have significant implications for the various strategic modelling frameworks informing current disease control and eradication efforts [18,19], as they suggest that control at higher temperatures might be more feasible than currently predicted. The results also add complexity to studies investigating the possible effects of climate warming [20], as increases in temperature need not simply lead to increases in transmission.
